# Myo/Nog Cells: Targets for Preventing the Accumulation of Skeletal Muscle-Like Cells in the Human Lens

**DOI:** 10.1371/journal.pone.0095262

**Published:** 2014-04-15

**Authors:** Jacquelyn Gerhart, Marvin Greenbaum, Victoria Scheinfeld, Paul FitzGerald, Mitchell Crawford, Arturo Bravo-Nuevo, Meghan Pitts, Mindy George-Weinstein

**Affiliations:** 1 Lankenau Institute for Medical Research, Wynnewood, Pennsylvania, United States of America; 2 Lankenau Medical Center, Wynnewood, Pennsylvania, United States of America; 3 Department of Cell Biology and Human Anatomy, School of Medicine, University of California Davis, Davis, California, United States of America; Case Western Reserve University, United States of America

## Abstract

Posterior capsule opacification (PCO) is a vision impairing condition that arises in some patients following cataract surgery. The fibrotic form of PCO is caused by myofibroblasts that may emerge in the lens years after surgery. In the chick embryo lens, myofibroblasts are derived from Myo/Nog cells that are identified by their expression of the skeletal muscle specific transcription factor MyoD, the bone morphogenetic protein inhibitor Noggin, and the epitope recognized by the G8 monoclonal antibody. The goal of this study was to test the hypothesis that depletion of Myo/Nog cells will prevent the accumulation of myofibroblasts in human lens tissue. Myo/Nog cells were present in anterior, equatorial and bow regions of the human lens, cornea and ciliary processes. In anterior lens tissue removed by capsulorhexis, Myo/Nog cells had synthesized myofibroblast and skeletal muscle proteins, including vimentin, MyoD and sarcomeric myosin. Alpha smooth muscle actin (α-SMA) was detected in a subpopulation of Myo/Nog cells. Areas of the capsule denuded of epithelial cells were surrounded by Myo/Nog cells. Some of these cell free areas contained a wrinkle in the capsule. Depletion of Myo/Nog cells eliminated cells expressing skeletal muscle proteins in 5-day cultures but did not affect cells immunoreactive for beaded filament proteins that accumulate in differentiating lens epithelial cells. Transforming growth factor-betas 1 and 2 that mediate an epithelial-mesenchymal transition, did not induce the expression of skeletal muscle proteins in lens cells following Myo/Nog cell depletion. This study demonstrates that Myo/Nog cells in anterior lens tissue removed from cataract patients have undergone a partial differentiation to skeletal muscle. Myo/Nog cells appear to be the source of skeletal muscle-like cells in explants of human lens tissue. Targeting Myo/Nog cells with the G8 antibody during cataract surgery may reduce the incidence of PCO.

## Introduction

Posterior capsule opacification (PCO) is a vision impairing condition that arises in some patients following cataract surgery [1], [2]. Visual acuity is compromised by the formation of Elschnig pearls that consist of differentiating lens cells (regenerative PCO) and the emergence of myofibroblasts that migrate onto the lens capsule and deposit extracellular matrix (fibrotic PCO) [3]. The fibrotic form of PCO has been attributed to lens epithelial cells that undergo an epithelial to mesenchymal transition (EMT) and a transdifferentiation to myofibroblasts [2], [4]. Several families of molecules have been implicated in the emergence of myofibroblasts in lens tissue [43], including transforming growth factor beta (TGF-β) that induces an epithelial to mesenchymal transition (EMT), cell migration, synthesis of alpha smooth muscle actin (α-SMA), contraction and production of extracellular matrix in anterior and posterior lens tissue [4]–[18]. Contractions of myofibroblasts produce folds and wrinkles in the thick basement membrane surrounding the lens called the capsule [19].

Myofibroblasts in the chick embryo lens originate from Myo/Nog cells that are incorporated into the eye during early stages of development [20]–[22]. Myo/Nog cells, which exist at low frequency in many tissues, are identified by their expression of mRNA for the skeletal muscle specific transcription factor MyoD, the bone morphogenetic protein (BMP) inhibitor Noggin and the cell surface molecule recognized by the G8 monoclonal antibody (mAb) [20], [21], [23]–[27]. Expression of MyoD is the hallmark of Myo/Nog cells’ commitment to the skeletal muscle lineage, while their release of Noggin is critical for modulating BMP signaling, morphogenesis and differentiation [20], [21], [26], [28]. Depletion of Myo/Nog cells in the blastocyst results in severe malformations of the body wall, central nervous system and the eyes due to de-regulated BMP signaling [20], [21], [26].

In addition to their role as the primary producer of Noggin, Myo/Nog cells react to a perturbation in homeostasis in multiple tissues [22], [26], [27]. The propensity of Myo/Nog cells to respond to wounding reflects, in part, their innate capacity for migration and expression of muscle proteins [20]–[22], [24], [25], [29]. When removed from embryonic and fetal tissues and cultured in serum-free medium, they translate MyoD mRNA and undergo terminal skeletal muscle differentiation [24], [25], [28], [29]. *In vivo*, Myo/Nog cells do not appear to translate MyoD mRNA or synthesize sarcomeric proteins under homeostatic conditions [21], [25], [30]. However, when activated by apoptotic cells, epidermal abrasion, tumorigenesis, or incisions in embryonic lens tissue, Myo/Nog cells rapidly increase in number and migrate to the wound [22], [26], [27].

The goals of this study were to determine whether the human lens contains Myo/Nog cells and characterize their behavior in anterior lens tissue removed by capsulorhexis during cataract surgery. The effect of Myo/Nog cell depletion on the accumulation of myofibroblasts in anterior lens tissue was tested *in vitro*.

## Materials and Methods

### Human Anterior Segments

Human anterior segments from three donors were obtained through the National Disease Research Interchange (Philadelphia, PA, USA). Lens sections from an additional three donors were acquired from Excalibur Pathology, Inc. (Oklahoma City, OK, USA). The age of the donors ranged from 52 to 96 years old. Five eyes were procured 3–8 hours postmortem. The sixth eye was procured 13 hours postmortem. Eyes were immediately placed in fixative and shipped the same day. Lenses from one donor were fixed in 4% paraformaldehyde for approximately 24 hours, cut in quarters and embedded in OCT compound for cryosectioning. Lenses from the other five donors were fixed in a modified Davidson’s fixative containing 14% ethyl alcohol, 14% formalin and 6.25% glacial acetic acid (Excalibur Pathology, Inc., USA) and embedded in paraffin. This fixation method produced preservation of lens morphology superior to that of paraformaldehyde. Tissue was sectioned at 10 µm. Some sections were stained with Hematoxylin 7211 and Eosin-Y (Richard-Allan Scientific, Kalamazoo, MI, USA).

### Human Anterior Lens Tissue

Anterior lens tissue was removed by capsulorhexis from 283 patients during cataract surgery. The patients ranged from 55 to 91 years old. Approximately 2/3 of the patients were female. The most common cataract subtype was nuclear sclerosis, which in the majority of cases, was accompanied by cortical and less commonly, posterior subcapsular cataracts. The tissue was fixed in 2% paraformaldehyde within 10 minutes of its removal from 66 patients. Each whole piece of anterior lens tissue was cut in half before processing for histological analysis. Other pieces of unfixed anterior lens tissue were cultured by a modification of the methods of Wormstone et al. [31] and Ishizaki et al. [32]. Four edges of the capsule were pressed to a 35 mm tissue culture dish. Lens tissue was cultured in serum and protein-free DMEM/F12 medium (GIBCO/Life Technologies, Grand Island, NY, USA) at 37°C in 5% CO_2_ in air.

### *In Situ* Hybridization and Immunofluorescence Localization

Sections of the anterior segment or anterior lens tissue removed during cataract surgery were examined for the expression of the G8 epitope and mRNAs for MyoD and Noggin by incubating with the G8 IgM MAb [25] and goat anti-mouse IgM µ chain antibodies conjugated with DyLight 488 (Invitrogen/Molecular Probes, Eugene, OR, USA), followed by incubation in Cy3 labeled 3DNA™ dendrimer nanoparticles (Genisphere, LLC, Hatfield, PA, USA) [33]. The following anti-sense sequences were conjugated to 3DNA: human MyoD1 (NM_002478.4∶5′-CTGTCCGGCCTGATTTGT GGTTAAGGA-3′) and mouse Noggin (NM_008711.2∶5′-TCTCGTTCAGATCC TTCTCCTTAGGGTCAAA-3′) [34], [35]. The sequence to mouse Noggin was 94% homologous to human Noggin (29 out of 31 bases) [36] and showed the same co-localization pattern with the G8 mAb in murine and human tissues [27].

Sections of the anterior segment and anterior lens tissue were double labeled with the G8 IgM mAb to tag Myo/Nog cells, and IgG mAbs to vimentin (AMF-17b) [37], alpha smooth muscle actin (α-SMA) (directly conjugated with fluorescein; Sigma-Aldrich, St. Louis, MO, USA) (markers of myofibroblasts), MyoD1 protein (NCL-MyoD1; Novocastra Labs Ltd, UK), slow sarcomeric myosin (A4.951) [38], neonatal and adult sarcomeric myosin heavy chain (MF30) [39], the skeletal muscle specific, T-tubule associated 12101 antigen (12101) [40] and cardiac and skeletal muscle troponin T (CT3) [41] (markers of striated muscle) by previously described methods [23], [25]. Double labeling was also performed with the G8 mAb and goat polyclonal antibodies to Noggin (AF719; R&D Systems, Minneapolis, MN, USA) and rabbit polyclonal antibodies to filensin [42] and CP49 [43], [44]. Controls for non-specific staining included the E12 IgM mAb [29], 2H3 IgG mAb to neurofilament protein [45] and a goat polyclonal antiserum to the homeobox protein LBX1 expressed in the central nervous system and some developing muscles [46] (Santa Cruz Biotechnology, Dallas, TX, USA). Monoclonal antibodies to vimentin, sarcomeric myosins, the 12101 antigen, troponin T and neurofilament protein were obtained from the Developmental Studies Hybridoma Bank (developed under the auspices of the NICHD and maintained by the University of Iowa, Dept. of Biology, Iowa City, IA, USA).

Primary antibodies were visualized with species- and subclass-specific fluorescent secondary antibodies, including goat anti-mouse or anti-rabbit IgG, goat anti-mouse IgM µ chain and donkey anti-goat IgG conjugated with DyLights 488 or 549 (Jackson ImmunoResearch, West Grove, PA, USA). Nuclei were stained with Hoechst dye 33258 (Sigma-Aldrich). Labeling was analyzed with the Nikon Eclipse E800 epifluorescence microscope equipped with the Evolution QE Optronics video camera and Image Pro Plus image analysis software program (Media Cybernetics, Rockville, MD, USA), and Nikon Eclipse Ti Confocal microscope and NIS-Elements software. Figures were annotated and uniformly adjusted for brightness and contrast with Adobe Photoshop 6.0.

The percentage of labeled cells in anterior lens tissue was determined by counting the total numbers of stained and unstained cells in 20 consecutive fields across the entire tissue. The number of cells in 20 fields varied between 1,124 and 2,284. The accuracy of this sampling method was determined by comparing the percentage of labeled cells in 20 fields to the percentage in the entire tissue. The values for each combination of antibodies used for double labeling are the mean ± standard deviation of anterior lens tissue from different patients.

### Depletion of Myo/Nog Cells in Anterior Lens Cultures

Cultures containing anterior lens tissue were incubated in Hanks buffered saline containing 0.1% bovine serum albumen (Sigma-Aldrich) and the G8 mAb or baby rabbit complement (Cedar Lane, Inc., Hornby, Ontario, Canada, USA) alone, or a pre-mixed solution of G8 and complement 20 hours after plating [21]. Explants were stained with a fluorescent secondary antibodies to visualize the G8 mAb, and terminal deoxynucleotidyl transferase dUTP nick end labeling (TUNEL) reagents (Roche Diagnostics, Mannheim, Germany) five hours after treatment [26]. Other treated explants were incubated in culture medium for an additional 4 days and then stained with antibodies to muscle and beaded filament lens proteins.

### Treatment of Wounded Anterior Lens Tissue with TGF-β

Whole pieces of anterior lens tissue were cut in half and pressed to a culture dish. In some cultures, tissue was treated with the G8 mAb and complement to deplete Myo/Nog cells immediately after plating. A scratch wound was produced by gently abrading the epithelium with a knife by modification of the method of Wormstone et al. [4], [14]. The location of the wound was marked at the edge of the tissue with a dissecting needle. Following wounding, explants were incubated in one ml of DMEM/F12 medium alone or DMEM/F12 containing 10 ηg/ml human recombinant TGF-β2 (Sigma-Aldrich and R&D Systems, Inc., Minneapolis, MN, USA) or human recombinant TGF-β1 (Sigma-Aldrich). Some cultures were treated simultaneously with TGF-β1 or −β2 and 10 ηg/ml human recombinant Noggin (PeproTech, Inc., Rocky Hill, NJ, USA). Media were replenished 48 hours later. Cultures were fixed 48 or 120 hours following the initiation of treatment.

### Statistical Analyses

The two-tailed Student’s t-test was used to compare the percentages of cells labeled with antibodies to G8, α-SMA, MyoD, myosin, filensin and CP49 in treated and control cultures.

### Use of Human Tissue and Ethics Statement

This research followed the tenets of the Declaration of Helsinki. Written informed consent was obtained from the subjects following an explanation of the study. The project was approved by the Institutional Review Board of Main Line Hospitals.

## Results

### Myo/Nog Cells are Present in the Anterior Segment of the Human Eye

**Figure 1:**
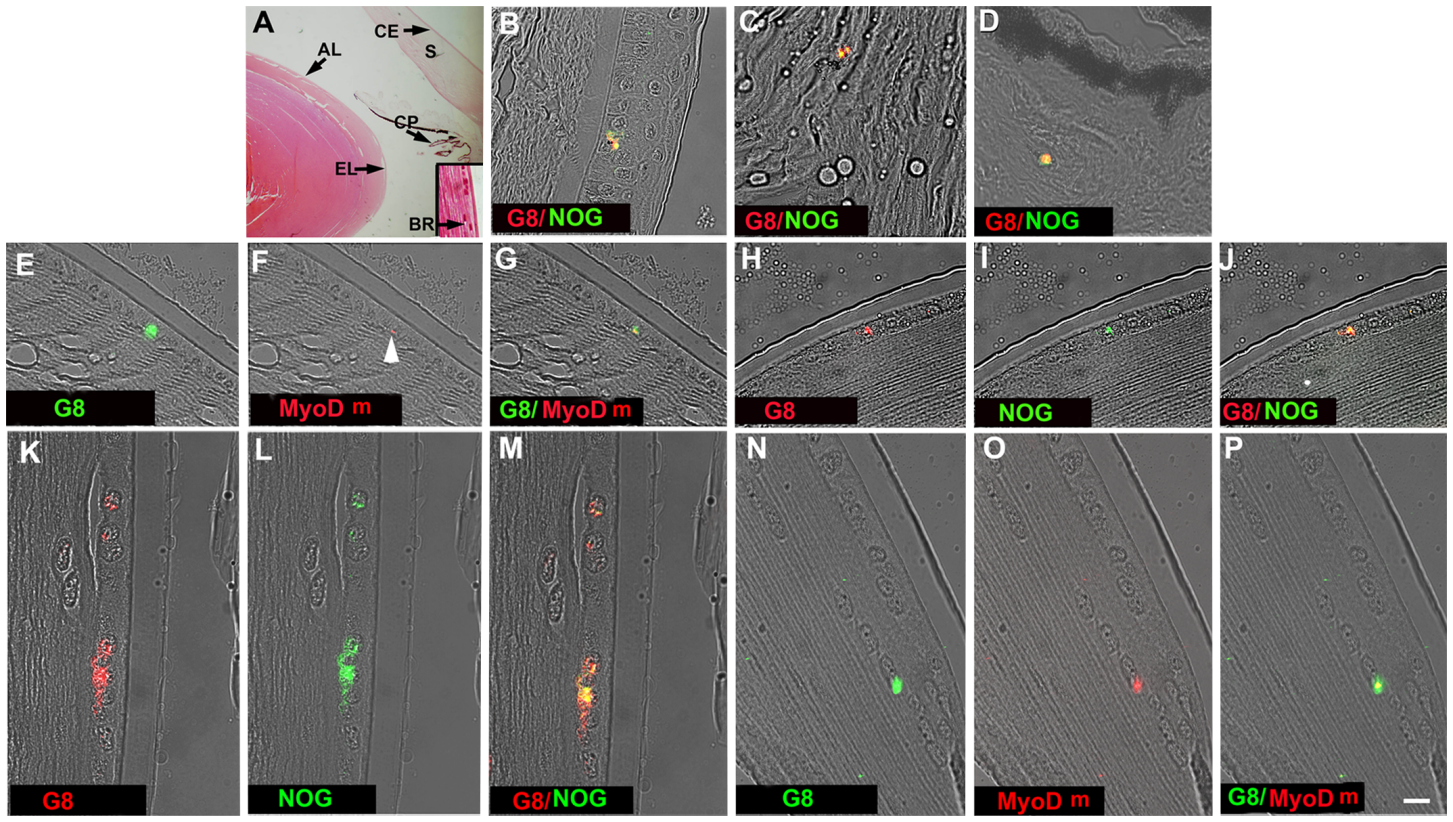
Distribution of Myo/Nog cells in the human anterior segment. Tissue sections through the anterior segment were stained with H&E (A) or double labeled with G8 and dendrimers to MyoD mRNA (MyoD m) or an antibody to Noggin (NOG) (B–P). The primary antibodies and colors of the fluorescent dendrimers and secondary antibodies are indicated in each photograph. Photographs are the merged images of DIC and fluorescence. Overlap of green and red appears yellow in merged images. Myo/Nog cells labeled for G8, MyoD mRNA and Noggin protein were present in the anterior (E–J), equatorial (K–M) and bow regions (N–P) of the lens, corneal epithelium (B), corneal stroma (C) and ciliary processes (D). AL = anterior lens, EL = equatorial lens, BR = bow region of the lens shown in the inset in A, CP = ciliary process, CE = corneal epithelium, S = corneal stroma. Bar = 135 µm in A and 9 µm in B–I.

Tissue sections from the anterior segment of six donors were probed for G8, MyoD mRNA and Noggin to determine whether Myo/Nog cells are present in the human lens. None of the donors were reported to have had a history of lens disease. *In situ* hybridization was carried out with Cy3 labeled 3DNA dendrimers that are extremely sensitive and precise reagents for localizing mRNA in single cells in fresh and sectioned tissue [20], [21], [23], [25]–[27], [33]. Double labeling with the G8 mAb and dendrimers to MyoD mRNA or an antibody to Noggin revealed the presence of small numbers of Myo/Nog cells in the lens, epithelial and stromal layers of the cornea, and ciliary processes (Figure 1). Each 10 µm section of the whole lens contained an average of 4±4 (n = 60) Myo/Nog cells among the lens epithelial cells. Of the total cells in the lens labeled with the G8 mAb, 28%, 40% and 31% were present in the anterior, equatorial and bow regions, respectively. Single or clusters of Myo/Nog cells were found in each of these regions, although they were not visible in all three areas in every section (Figure 1E–P). Only one Myo/Nog cell was found between the cortical fibers. These results are consistent with our findings in the chick embryo [20] and adult mice and rabbits (unpublished data) in which the eyes were removed and fixed immediately upon death.

### Myo/Nog Cells in Anterior Lens Tissue Removed by Capsulorhexis Express Markers of Myofibroblasts and Skeletal Muscle Cells

**Figure 2:**
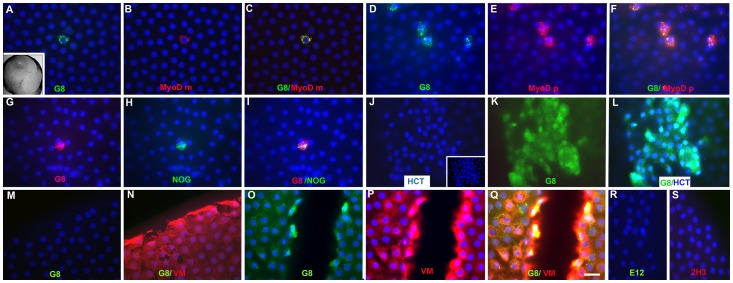
Distribution of Myo/Nog cells in human anterior lens tissue removed during cataract surgery. A low magnification DIC image of anterior lens tissue fixed after capsulorhexis is shown in the inset in A. Tissue was double labeled with the G8 mAb and dendrimers to MyoD mRNA (MyoD m) or antibodies to MyoD protein (MyoD p), Noggin (NOG) or vimentin (VM). The primary antibodies and colors of the fluorescent dendrimers and secondary antibodies are indicated in each photograph. Unmerged images precede the merged images shown in C, F, I, L and Q. Overlap of green and red appears yellow in merged images. Nuclei were stained with Hoechst dye (HCT) (blue). Anterior lens tissue contained single, small groups or large clusters of Myo/Nog cells throughout the epithelium (A–L). Photographs in J-L illustrate a cluster of G8+ cells lying on the apical surface of lens epithelial cells. The underlying layer of nuclei in the same field is shown in the inset in J. G8+ cells were present along the cut edge of some (O) but not all samples (M). Vimentin staining was most intense at the periphery of the tissue (N and P). Tissue incubated with the E12 IgM or 2H3 IgG and their respective secondary antibodies lacked fluorescence (R and S). Bar = 5 mm in the inset in A and 9 µm in the other photomicrographs.

Anterior lens tissue, which is more readily available than whole lenses and can be obtained within minutes of surgery, was used to explore the properties of Myo/Nog cells in the human lens. Every piece of anterior lens tissue that was fixed within 10 minutes of capsulorhexis contained a small population of cells labeled with the G8 mAb (3% ±2, number of patients (n = 66) (Figure 2). MyoD mRNA was detected in practically all G8+ cells (98% ±2, n = 4) (Figure 2A–C), and only a few MyoD mRNA+ cells lacked staining for G8 (4% ±4, n = 4). Most Myo/Nog cells had translated MyoD mRNA into protein (Table 1; Figure 2D–F). Noggin was localized in 99% of the G8+ cells (Table 1; Figure 2G–I).

**Table 1:** Myo/Nog cells in anterior lens tissue are immunoreactive for proteins found in skeletal muscle and lens tissue.

Antibody	% Ab Positive	% Ab Positive with G8	% G8 Positive with other Ab
MyoD	2±2	100	94±12
Noggin	3±2	99±1	99±2
Vimentin	92±10	6±2	100
α-SMA	1±1	30±26	12±11
Sarcomeric Myosin	4±1	93±16	90±19
12101	4±2	81±27	90±12
Troponin T	2±1	100	54±25

Anterior lens tissue was fixed within 10 minutes of capsulorhexis and double labeled with G8 and other antibodies (Ab) and species and subclass specific fluorescent secondary antibodies. % Ab Positive = number of fluorescent cells ÷ total number of cells in 20 fields X 100. % Ab Positive with G8 =  number of antibody positive cells co-labeled with G8 ÷ total antibody positive cells X 100. Percent G8 Positive Cells with Other Ab = number of G8 positive cells co-labeled with the other antibody ÷ total number of G8 positive cells X 100. Four cultures were scored for each pair of antibodies except G8+ Noggin (n = 9) and G8+ α-SMA (n = 10).

Single or small clusters of 2–4 Myo/Nog cells appeared to be randomly distributed among the lens epithelial cells (Fig. 2A–I). Approximately 35% of the anterior lenses contained clusters ranging from 4–40 Myo/Nog cells lying on the apical surface of the epithelial cells (Figure 2J–L). The presence of apical clusters did not appear to correlate with the age (55–89 years), cataract subtype (nuclear sclerosis with or without cortical and/or posterior subcapsular) or grade (2–4) of the donor. Most lenses lacked G8+ cells along the incision created during the initiation of capsulorhexis (Figure 2M); however, in 15% of the specimens, G8+ cells were intermittently aligned along the incisional edge (Figure 2O). By contrast, all cells along the entire periphery of the tissue were intensely labeled with an antibody to the intermediate filament protein vimentin, (Figure 2N and P). Less intense vimentin staining was observed throughout the epithelium (Figure 2N and P). The specificity of staining with the G8 and vimentin antibodies along the edge of the tissue was demonstrated by the absence of fluorescence when the tissue was incubated with the E12 IgM mAb, 2H3 IgG mAb and their respective secondary antibodies (Figure 2R and S) or secondary antibodies alone (not shown). Thus, in some samples, Myo/Nog cells appear at the incisional border of the tissue.

**Figure 3:**
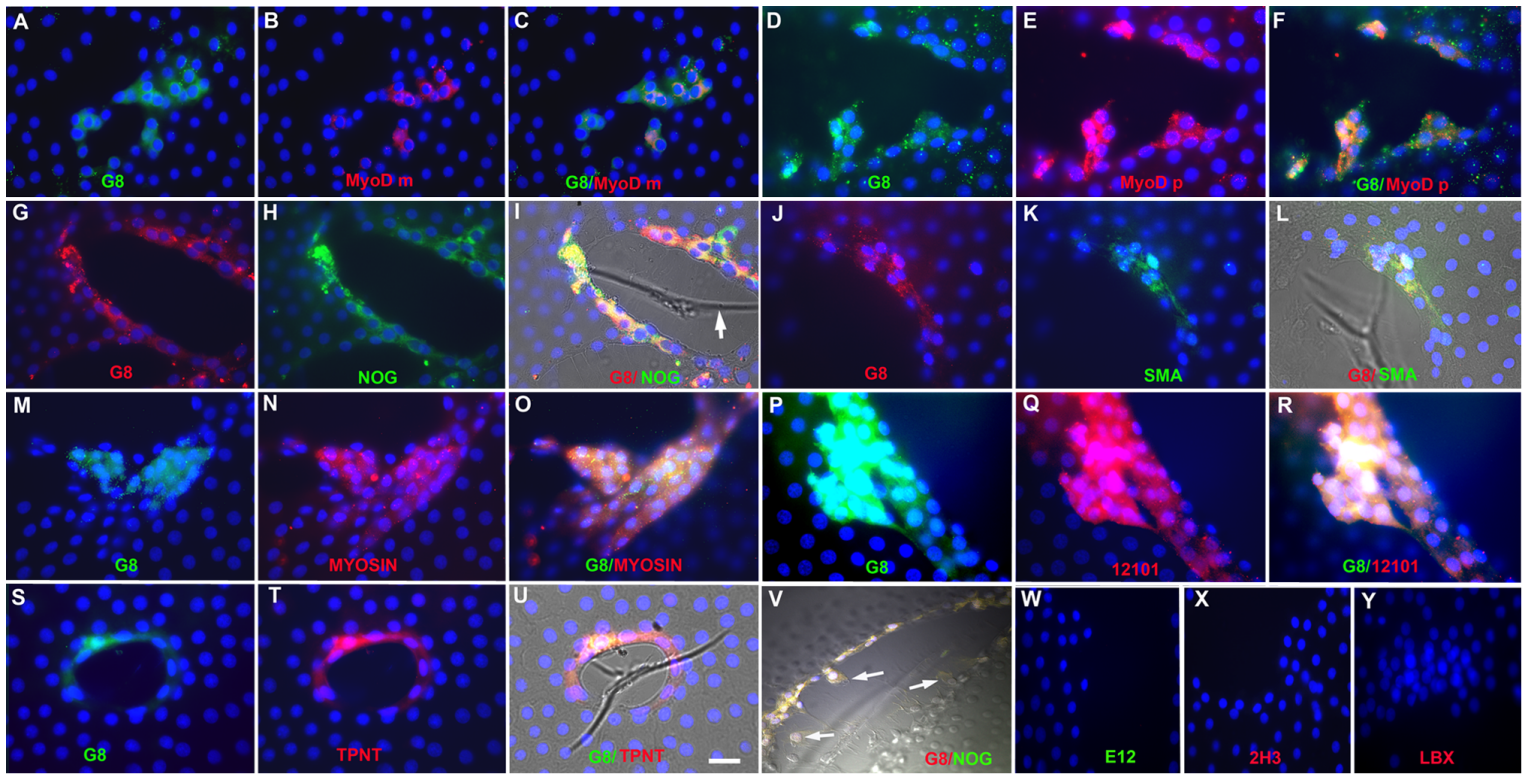
Myo/Nog cells in human anterior lens tissue express muscle proteins. Anterior lens tissue fixed after capsulorhexis was double labeled with the G8 mAb and dendrimers to MyoD mRNA (MyoD m) or antibodies to MyoD protein (MyoD p), Noggin (NOG), α-SMA, sarcomeric myosin heavy chain (MYOSIN), the skeletal muscle specific 12101 antigen (12101) and troponin T (TPNT). The primary antibodies and colors of the fluorescent dendrimers and secondary antibodies are indicated in each photograph. Overlap of green and red appears yellow in merged images. Nuclei were stained with Hoechst dye (blue). Panels I, L, U and V are quadruple merged images of DIC and fluorescence showing wrinkles in the capsule (arrow in I). G8+ cells co-stained for MyoD mRNA, Noggin and muscle proteins surrounded cell free areas of the capsule (A–V). Some Myo/Nog cells had migrated onto the capsule (arrows in V). Tissue incubated with the E12 or 2H3 mAbs, or an antiserum to LBX1 and their respective secondary antibodies, lacked fluorescence (W–Y). Bar = 9 µm.

The state of differentiation of Myo/Nog cells in the lens was determined by screening for markers of myofibroblasts and skeletal muscle cells. Low numbers of cells throughout the lens tissue were labeled with antibodies to muscle proteins (Table 1; Figure 3). Whereas only a small percentage of the G8+ cells contained detectable levels of α-SMA, most or all G8+ cells had synthesized MyoD, sarcomeric myosins, the skeletal muscle specific, T-tubule associated 12101 molecule and troponin T (Table 1), indicating that Myo/Nog cells synthesize proteins characteristic of skeletal muscle.

Approximately 76% of the anterior lenses contained small areas in which the epithelial cells had been denuded from the capsule. These areas were distant from the edges of the tissue pressed against the tissue culture dish. A wrinkle was present in the capsule in at least one of these cell free areas in approximately one third of the samples (Figure 3I, L, U and V). Myo/Nog cells had formed a thickened rim around these cell free areas (Figure 3A–V). Most of the G8+ cells surrounding the bare areas of capsule expressed MyoD mRNA and stained with antibodies to MyoD protein, Noggin, α-SMA, vimentin (not shown), sarcomeric myosin, 12101 and troponin T (Figure 3A–V). The specificity of antibody binding in Myo/Nog cells was demonstrated by the lack of staining around the edges of cell free areas following incubation with the E12 and 2H3 mAbs, the LBX1 goat polyclonal antiserum and their respective secondary antibodies (Figure 3W–Y), or secondary antibodies alone (not shown). Although Myo/Nog cells expressed sarcomeric proteins, they did not appear striated. Some Myo/Nog cells were present on the capsule within the cell free areas and had extended processes towards the wrinkle (Figure 3V).

### Depletion of Myo/Nog Cells in Cultures of Anterior Lens Tissue Prevents the Accumulation of Cells Expressing Muscle Proteins

**Figure 4:**
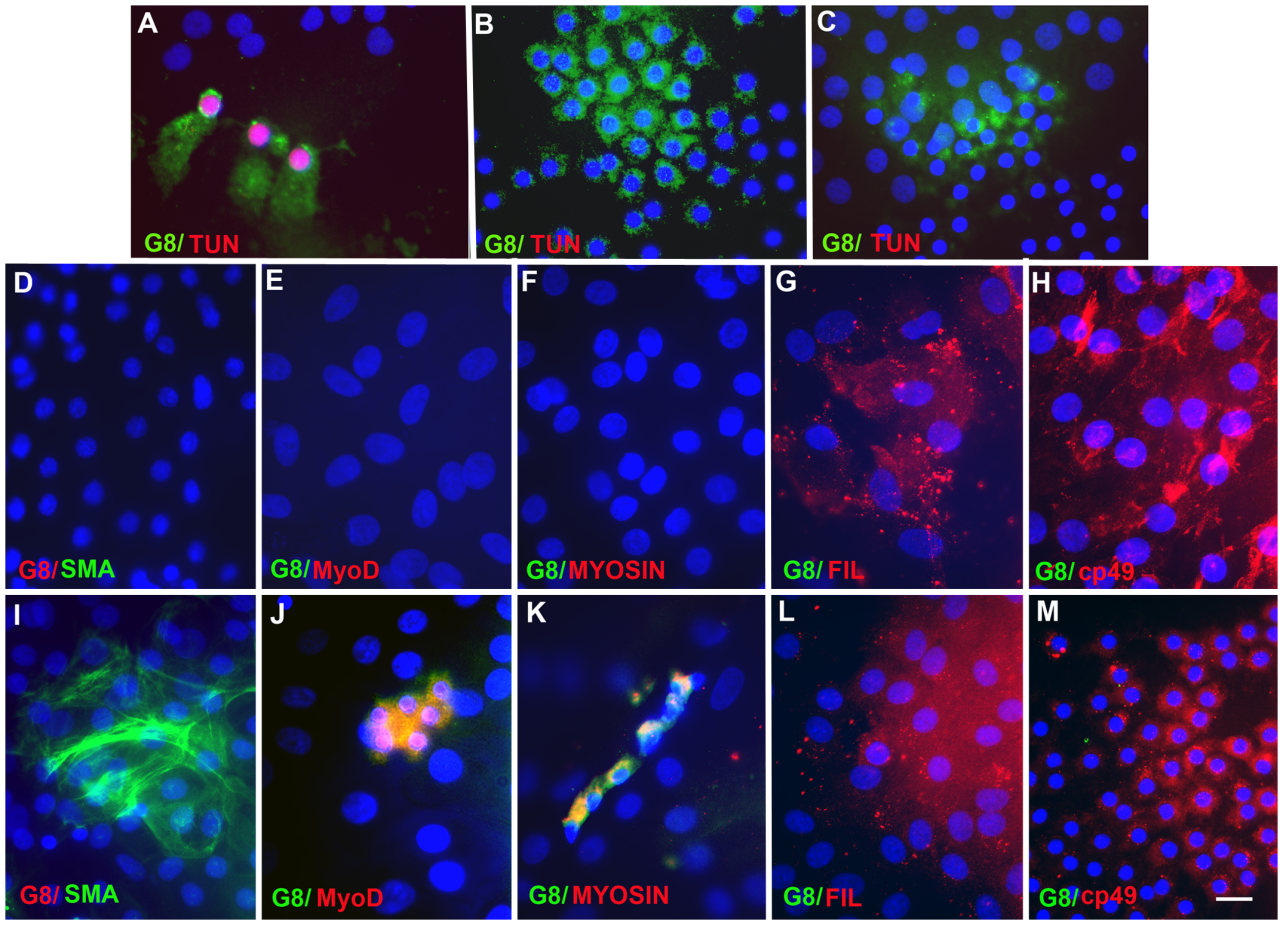
Effects of targeting Myo/Nog cells with the G8 mAb and complement in anterior lens explants. Explants of lens tissue were incubated with the G8 mAb and complement (A, D–H), G8 only (B, I–M) or complement only (C). Five hours later, the tissue was double labeled with the G8 mAb (green) and TUNEL reagents (red). Nuclei were stained with Hoechst dye. G8+, but not G8- cells, were TUNEL+ following treatment with G8 and complement (A). G8+ cells were not TUNEL+ when treated with G8 or complement alone (B and C). Five days following ablation, explants were double labeled with antibodies to G8 and MyoD, α-SMA, sarcomeric myosin heavy chain (MYOSIN), filensin and cp49. The colors of the fluorescent secondary antibodies are indicated in each photograph. Overlap of green and red appears yellow in merged images. Ablation of Myo/Nog cells prevented the accumulation of G8+, MyoD+ and α-SMA+ cells, but not filensin+ and cp49+ cells (D–H). Both muscle and beaded filament proteins were detected in explants treated with the G8 mAb alone (I–M). Bar = 9 µm.

The effect of depleting Myo/Nog cells on the accumulation of muscle cells in explants of anterior lens tissue was determined by lysing cells that bound the G8 mAb with complement [21]. Five hours following treatment with G8 and complement, 99% ±1 of the G8+ cells, but only 3% ±3 of the G8- cells (n = 5) were undergoing apoptosis, as indicated by TUNEL staining (Figure 4A). In cultures treated with the G8 mAb or complement only, 2% ±2 and 5% ±8 of the G8+ and G8- cells, respectively, were TUNEL+ (n = 6) (Figure 4B and C). This experiment demonstrates that Myo/Nog cells are specifically targeted when lens explants are treated with G8 and complement.

Four days following Myo/Nog cell depletion, cultures were labeled with antibodies to G8 and muscle proteins. Treatment with both G8 and complement eliminated G8+ cells in 12 out of 16 cultures and left only a few G8+ cells in the remaining cultures (Table 2, Figure 4D–H). The percentage of α-SMA+ cells was reduced approximately 6-fold following treatment with G8 and complement (Table 2, Figure 4E) and only 1% ±3 of the α-SMA+ cells were co-labeled with the G8 mAb. Complete elimination of G8+ cells in 5-day cultures was accompanied by an absence of MyoD+ and sarcomeric myosin+ cells (Figure 4E and F). By contrast, ablation of Myo/Nog cells did not prevent the accumulation of cells immunoreactive for the beaded filament proteins filensin and CP49 that are synthesized in differentiating lens cells (Table 2, Figure 4G and H). Control cultures treated with G8 (Table 2, Figure 4I–M) or complement alone contained cells with muscle and beaded filament proteins (Table 2). These experiments demonstrate that Myo/Nog cells are not replenished following treatment with the G8 mAb and complement, and the depletion protocol inhibits the accumulation of myogenic cells.

**Table 2:** Effect of depleting Myo/Nog cells on the accumulation of cells immunoreactive for muscle and beaded filament proteins in anterior lens cultures.

	G8 mAb Tx	Comp Tx	G8 mAb+Comp Tx
% G8+	11±13 (19)	18±12 (4)	0.1±0.1 (16)
% α-SMA+	22±15 (4)	24±14 (4)	4±4 (6)
% MyoD+	5±5 (5)	ND	0 (6)
% Myosin+	5±1 (4)	ND	0 (4)
% Filensin+	18±8 (4)	ND	12±9 (4)
% CP49+	12±5 (4)	ND	24±12 (4)

Anterior lens tissue was incubated with the G8 mAb or complement (Comp) alone, or G8 and complement, 20 hours after plating. Cultures were double labeled with antibodies to G8 and α-SMA, MyoD, sarcomeric myosin, filensin or CP49 on the fifth day in culture. Values are the mean ± standard deviation of the number of antibody-positive cells ÷ total cells X 100. The number of cultures scored is indicated in parentheses. No significant differences were found between the percentages of G8+ or α-SMA+ cells treated with either the G8 mAb or complement alone. Significant differences were found in cultures treated with either the G8 mAb or complement alone and G8+ complement in the percentages of G8+ (0.003 and 0.0001, respectively), α-SMA+ (0.02 and 0.006, respectively), MyoD+ (0.0001) and myosin+ cells (0.0001). No significant differences were found between the percentages of filensin+ and CP49+ cells in cultures treated with G8 or G8+ complement.

### TGF-β Does Not Induce Expression of Skeletal Muscle Proteins in the Absence of Myo/Nog Cells

**Figure 5:**
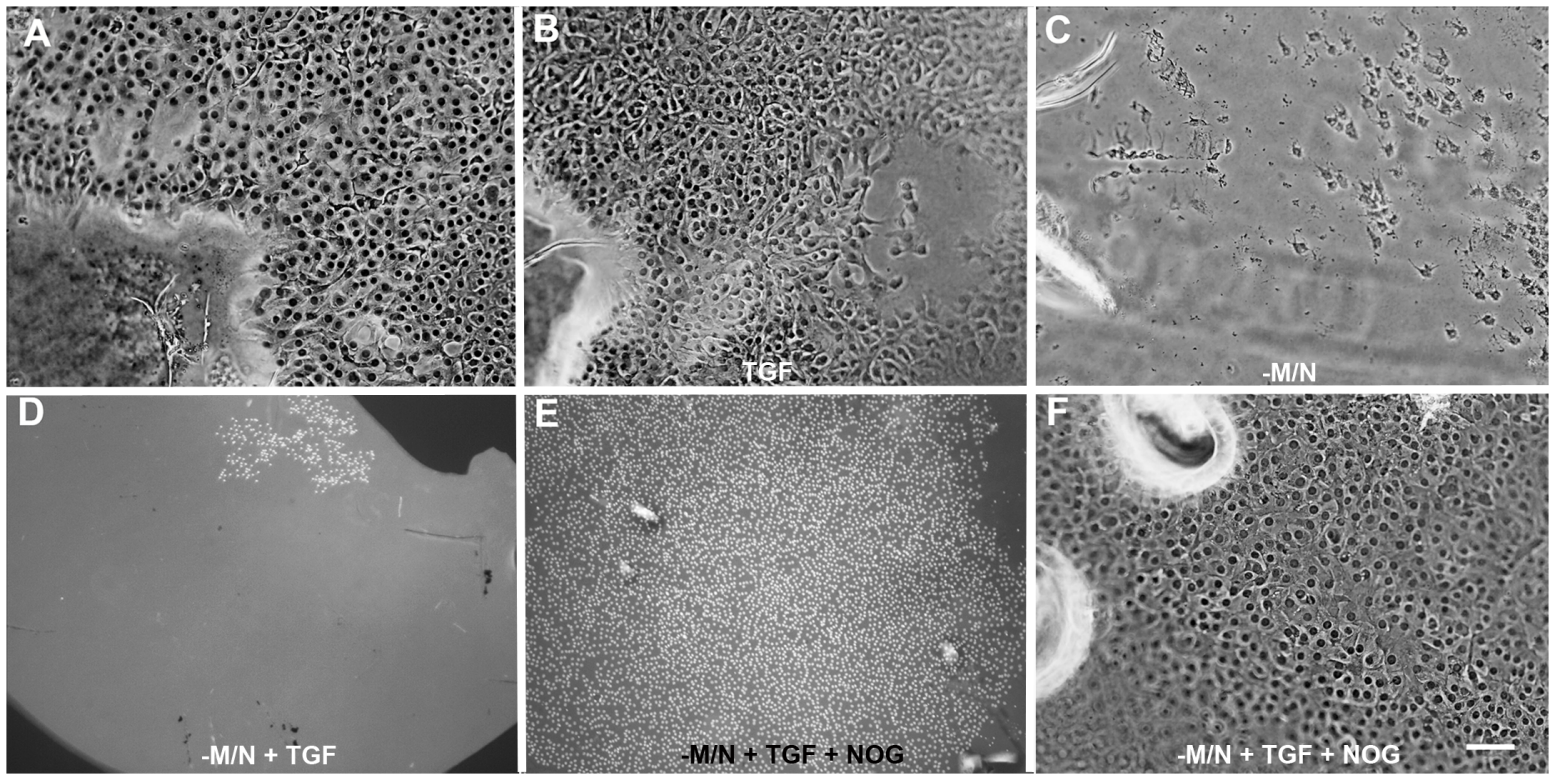
Effects of Myo/Nog cell depletion and TGF-β2 on wound healing in anterior lens explants. A scratch wound was created in explants of anterior lens tissue. Cells had populated the capsule denuded of cells within 48 hours of wounding (A). The wound was also covered with cells following treatment with TGF-β2 (B). Few cells were present in the wound of an explant depleted of Myo/Nog cells (-M/N) (C). The combination of wounding, depletion of Myo/Nog cells and treatment with TGF-β2 resulted in a loss of most cells from the capsule (D). Addition of Noggin (NOG) prevented cell loss resulting from depletion of Myo/Nog cells and treatment with TGF-β2 (E) and promoted wound healing (F). Bar = 27 µm in A-C and F, and 135 µm in D and E.

Anterior lens tissue depleted of Myo/Nog cells was further challenged to produce muscle cells by creating a scratch wound in the epithelium and incubating with TGF-β2 [4], [15], [47]–[49]. The wound healing response varied within and between treatment groups. Cells had partially filled in the wound within 48 hours in two out of three untreated explants (Figure 5A). Treatment with TGF-β2 for two days resulted in partial to complete wound closure in four out of 7 explants (Figure 5B). Only one out of five explants displayed partial wound healing following ablation of Myo/Nog cells (Figure 5C). The combination of Myo/Nog cell depletion, wounding and treatment with TGF-β2 resulted in the loss of approximately 50–90% of the cells by 48 hours (Figure 5D). We reasoned that the detrimental effect of TGF-β2 in explants lacking Myo/Nog cells could reflect the loss of Noggin. Indeed, addition of Noggin at the same time as TGF-β2 prevented cell loss throughout the explant in the absence of Myo/Nog cells, and the wounds were partially or completely filled in all five explants (Figure 5E and F).

**Figure 6:**
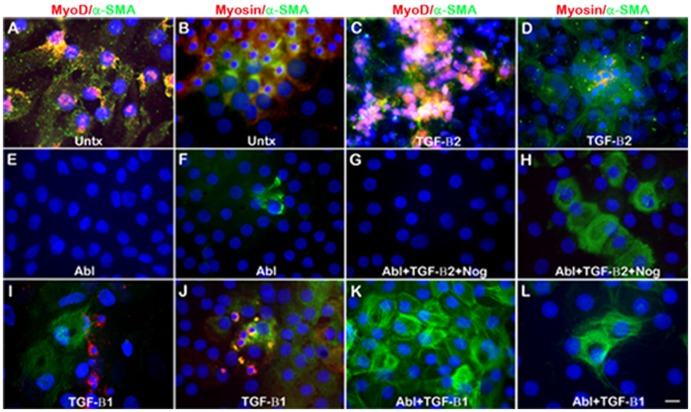
Effect of TGF-β on the accumulation of cells with muscle proteins following wounding and depletion of Myo/Nog cells. Anterior lens explants were wounded and incubated in medium alone (untx) or medium containing TGF-β2 or −β1. Myo/Nog cells were ablated (abl) in some explants prior to wounding. Cells were double labeled with antibodies to α-SMA (green) and MyoD or sarcomeric myosin (red). Nuclei were stained with Hoechst dye. Photographs were taken of the wounded area. Explants incubated in the presence or absence of TGF-β2 or −β1 contained α-SMA+, MyoD+ and myosin+ cells (A–D, I and J). Following depletion of Myo/Nog cells, α-SMA+ cells were less prevalent in the wounds of untreated (E and F) and TGF-β2 treated explants (G and H) than those treated with TGF-β1 (K). No MyoD+ or myosin+ cells were observed in the wounds following Myo/Nog cell depletion and incubation in the presence (G, H, K and L) or absence (E and F) of TGF-β2 or −β1. Bar = 9 µm.

By the fifth day after wounding, α-SMA+, MyoD+ and sarcomeric myosin+ cells were abundant in the wounds in untreated and TGF-β2 treated explants (Figure 6A–D). By contrast, no MyoD+ or myosin+ cells were detected within the wounds or throughout the tissue following depletion of Myo/Nog cells in the presence or absence TGF-β2 and Noggin (Figure 6E–H; Table 3). The percentages of α-SMA+ cells varied between explants (Table 3). Five out of six explants treated with TGF-β2 had less than 8% α-SMA+ cells; however in one explant, 60% were α-SMA+. The higher percentage of α-SMA+ cells in this tissue did not appear to correlate with age or the type or grade of cataract (age 70 with nuclear sclerosis (NS), grade 2 compared to those with ≤8% α-SMA+ cells, ages 65–86 with NS, NS with cortical (C) or NS with posterior subcapsular (PS) cataract, grades 2–4). The percentages of α-SMA+ cells remained consistently low following depletion of Myo/Nog cells in the presence or absence of TGF-β2 (Table 3).

**Table 3:** Effect of TGF-β on the accumulation of cells with muscle proteins in the presence and absence of Myo/Nog cells.

Treatment	% α-SMA+	% MyoD+	% Myosin+
Wd	6±6 (7)	4±3 (4)	11±9 (3)
Ab+Wd	2±2 (3)	0 (3)	0 (3)
Wd+TGF-β2	12±21 (7)	7±2 (3)	5±3 (4)
Ab+Wd+TGF-β2+Nog	3±4 (4)	0 (3)	0 (4)
Wd+TGF-β1	12±11 (5)	8±2 (4)	4±2 (3)
Ab+Wd+TGF-β1	62±43 (4)	0 (4)	0 (4)
Ab+Wd+TGF-β1+Nog	13±13 (4)	0 (4)	0 (4)

Some explants of anterior lens tissue were treated with the G8 mAb and complement to ablate (Ab) Myo/Nog cells. All explants were wounded (Wd) by scratching the epithelium. Explants were cultured in DMEM/F12 medium alone or medium containing TGF-β2 or −β1 with or without Noggin (Nog). Cells were double labeled with antibodies to α-SMA and MyoD or sarcomeric myosin. Values are the mean ± standard deviation of the number of antibody positive cells ÷ total cells X 100. The number of cultures scored is indicated in parentheses. Neither TGF-β2 nor −β1 stimulated the accumulation of MyoD+ or myosin+ cells following Myo/Nog cell ablation.

Unlike treatment with TGF-β2, cell loss was not observed in response to TGF-β1 following depletion of Myo/Nog cells (Figure 6I and J). In the presence of Myo/Nog cells, no significant differences were observed in the percentages of α-SMA+, MyoD+ or sarcomeric myosin+ cells with or without TGF-β1 or −β2, or between the two TGF-βs, with the exception of TGF-β1’s effect on increasing the size of the MyoD+ population (0.04). Following Myo/Nog cell depletion, the percentage of α-SMA+ cells was greater in response to TGF-β1 compared to TGF-β2 and Noggin (0.03); however, the results were highly variable (Table 3; Figure 6G, H, K and L). Large numbers of α-SMA+ cells did not appear to correlate with age or the type and grade of cataract (99% α-SMA+: ages 74 and 88, NS/C, grade 2/2 and NS/C/PS, grade 2/2/1, respectively; 22–27% α-SMA+: ages 83 and 74, NS/C, 2/2 and NS, 3, respectively). As was the case with TGF-β2, no MyoD+ or sarcomeric myosin+ cells were detected following depletion of Myo/Nog cells and treatment with TGF-β1 with or without Noggin (Figure 6K and L; Table 3). Combined, these results demonstrate that neither form of TGF-β stimulates the accumulation of cells expressing skeletal muscle proteins in the absence of Myo/Nog cells.

## Discussion

PCO occurs in approximately 10–20% in adults and 40–100% in children within two years of cataract surgery [50]–[53]. The incidence of PCO continues to rise three to six years post-operatively [52]. Although PCO may be ameliorated with Yttrium Aluminum Garnet (Nd:YAG) laser treatment, Nd:YAG:laser is not available worldwide and some patients develop serious complications from the procedure, including corneal edema and retinal detachment [1]. The incidence of PCO has been reduced or delayed by improvements in surgical and drug delivery techniques and intraocular lens composition and design [54], [55]; however, prevention of PCO remains an important goal in ophthalmology.

Fibrotic disease of the lens has been attributed to lens epithelial cells that undergo an EMT and a transdifferentiation to myofibroblasts [2], [4]. The capacity of epithelial cells in multiple tissues to undergo an EMT has been widely demonstrated in the developing embryo and during the invasion and metastasis of carcinomas [56]–[58]. However, multiple non-epithelial sources of myofibroblasts have been identified within the tumor stroma, wounds and fibrotic tissues [59]–[63], indicating that EMT is not necessarily required for the appearance of muscle-like cells in pathological environments.

We propose that a subpopulation of inherently myogenic cells within the lens contributes to the fibrotic form of PCO. In the embryo, these Myo/Nog cells originate in the epiblast, migrate and are integrated into the lens and retina during early stages of development [20], [21]. The origin of MyoD+/Noggin+/G8+ cells in the human lens is unknown, but their molecular signature and behaviors resemble those of Myo/Nog cells in avian, rodent and other human tissues [20]–[22], [27]. The premise that Myo/Nog cells in the human lens are a separate population from the lens fiber lineage is supported by the fact that G8+ cells specifically synthesize skeletal muscle proteins and their depletion prevents the emergence of skeletal muscle-like cells over time in culture without affecting the accumulation of cells immunoreactive for beaded filament proteins. These data are consistent with our previous findings demonstrating that Myo/Nog cells are a distinct subpopulation within the embryonic lens and they retain their myogenic properties in a variety of non-muscle tissues [20], [21], [25], [28].

The behavioral repertoire of Myo/Nog cells may be viewed as a continuum that is regulated by the environment. The parent Myo/Nog cell modulates the activities of BMPs in a variety of tissues and has repressed myogenic potential, as evidenced by the expression of MyoD mRNA without detectable levels of translation under homeostatic conditions [20], [21], [26], [28]. When isolated from the embryo and cultured in serum free medium, Myo/Nog cells translate MyoD mRNA, differentiate, fuse and assemble sarcomeres [24], [25], [29]. In response to a perturbation of homeostasis *in vivo*, Myo/Nog cells expand in number and rapidly appear at the wound [22], [26], [27]. Both Myo/Nog cells in the cataractous human lens (present study) and myofibroblasts [64]–[67] express multiple skeletal muscle genes, including MyoD, but they do not fuse or assemble sarcomeres [62], [64]–[67]. Even α-SMA, a commonly used marker for myofibroblasts and smooth muscle, is also expressed in embryonic and neonatal skeletal muscle [68]. Therefore, the development of Myo/Nog cells into myofibroblasts in response to injury appears to represent a partial, yet functional fulfillment of their myogenic potential.

The innate capacity of Myo/Nog cells to synthesize contractile proteins and their distribution around discontinuities in the epithelium are consistent with the interpretation that they were responsible for producing wrinkles in the capsule. Holes in the epithelium, called intercellular vesicles, were observed in sections of cataractous lenses [69], [70]. Gaps between human anterior epithelial cells can be produced from contractions induced by saline, acetylcholine or mechanical stimulation [71]. Importantly, expression of α-SMA is not required for contraction in lens cultures [49], suggesting that the expression of other muscle proteins, such as those synthesized by Myo/Nog cells, may be important mediators of capsular wrinkling.

While Myo/Nog cells in the human lens may have surrounded areas of the capsule denuded of epithelial cells and extended lamellipodia towards the wrinkle prior to surgery, their less common appearance at the cut edge of the tissue within 10 minutes of capsulorhexis may reflect a rapid migration to the wound, as observed in the chick embryo lens and murine skin [22], [27]. Another relatively rare phenomenon observed in this study was the appearance of Myo/Nog cells on the apical surface of lens epithelial cells. To our knowledge, cells interposed between the anterior epithelium and fibers *in vivo* have not been reported; however, in chick embryo capsular bag cultures, Myo/Nog cells do migrate on the surface of epithelial cells [22]. The occasional accumulation of Myo/Nog cells on the apical surface of lens epithelial cells and at the edge of the tissue may result from mechanical stretching that occurred during capsulorhexis and/or wounding of the epithelial sheet.

Depleting Myo/Nog cells in lens explant cultures significantly reduced the α-SMA+ population and eliminated MyoD+ and sarcomeric myosin+ cells. Addition of TGF-β1 or −β2 had variable effects on the accumulation of α-SMA+ cells that did not correlate with the type or grade of the cataract, and therefore, may reflect subtle differences in the tissue or its handling. Previous reports have documented a more consistent elevation of α-SMA in human lens cells by TGF-β [4], [14], [15], [49]. Differences between the results reported herein and previous studies may be attributed to the source of the tissue (anterior lens tissue, posterior capsular bag model or a lens epithelial cell line), method of measuring α-SMA (Western blot, RT-PCR or qualitative versus quantitative immunofluorescence microscopy), and length of exposure to TGF-βs (2–28 days).

While the effects of TGF-βs on α-SMA expression were variable, neither TGF-β1 nor −β2 stimulated the emergence of MyoD+ or sarcomeric myosin+ cells following depletion of Myo/Nog cells. These experiments further support the conclusion that Myo/Nog cells are a primary source of contractile myofibroblasts that express multiple skeletal muscle proteins following injury to lens tissue. Furthermore, Myo/Nog cells may modulate potential interactions between the BMP and TGF-β signaling pathways in the human lens, as evidenced by the ability of exogenous Noggin to prevent cell loss in response to TGF-β2.

This study provides proof of concept that Myo/Nog cells are potential targets in the human lens for reducing the fibrotic form of PCO and capsular wrinkling. Testing the safety and efficacy of Myo/Nog cell depletion in a preclinical model of cataract surgery may further establish the feasibility of immunotherapy to prevent secondary cataract formation. Depletion of Myo/Nog cells may also prove therapeutic in other tissues prone to fibrosis.

## References

[1] Awasthi N, Guo S, Wagner BJ (2009) Posterior capsular opacification: a problem reduced but not yet eradicated. Arch Ophthalmol 127: 555–562. 19365040 10.1001/archophthalmol.2009.3

[2] Wormstone IM, Wang L, Liu CS (2009) Posterior capsule opacification. Exp Eye Res 88: 257–269. 19013456 10.1016/j.exer.2008.10.016

[3] Raj SM, Vasavada AR, Joar K, Vasavada VA, Vasavada VA (2007) Post-operative capsular opacification: A review. Int J Biomed Sci 3: 13. PMC3614664 23675049

[4] Wormstone IM, Tamiya S, Anderson I, Duncan G (2002) TGF-beta2-induced matrix modification and cell transdifferentiation in the human lens capsular bag. Invest Ophthalmol Vis Sci 43: 2301–2308. 12091431

[5] Liu J, Hales AM, Chamberlain CG, McAvoy JW (1994) Induction of cataract-like changes in rat lens epithelial explants by transforming growth factor beta. Invest Ophthalmol Vis Sci 35: 388–401. 8112986

[6] Kurosaka D, Nagamoto T (1994) Inhibitory effect of TGF-beta 2 in human aqueous humor on bovine lens epithelial cell proliferation. Invest Ophthalmol Vis Sci 35: 3408–3412. 8056515

[7] Gordon-Thomson C, de Iongh RU, Hales AM, Chamberlain CG, McAvoy JW (1998) Differential cataractogenic potency of TGF-beta1, -beta2, and -beta3 and their expression in the postnatal rat eye. Invest Ophthalmol Vis Sci 39: 1399–1409. 9660488

[8] Saxby L, Rosen E, Boulton M (1998) Lens epithelial cell proliferation, migration, and metaplasia following capsulorhexis. Br J Ophthalmol 82: 945–952. 9828783 10.1136/bjo.82.8.945 PMC1722713

[9] Richiert DM, Ireland ME (1999) TGF-beta elicits fibronectin secretion and proliferation in cultured chick lens epithelial cells. Curr Eye Res 18: 62–71. 10075204 10.1076/ceyr.18.1.62.5393

[10] Lee EH, Joo CK (1999) Role of transforming growth factor-beta in transdifferentiation and fibrosis of lens epithelial cells. Invest Ophthalmol Vis Sci 40: 2025–2032. 10440257

[11] Marcantonio JM, Rakic JM, Vrensen GF, Duncan G (2000) Lens cell populations studied in human donor capsular bags with implanted intraocular lenses. Invest Ophthalmol Vis Sci 41: 1130–1141. 10752951

[12] Saika S, Okada Y, Miyamoto T, Ohnishi Y, Ooshima A, et al (2001) Smad translocation and growth suppression in lens epithelial cells by endogenous TGFbeta2 during wound repair. Exp Eye Res 72: 679–686. 11384156 10.1006/exer.2001.1002

[13] Saika S, Miyamoto T, Ishida I, Shirai K, Ohnishi Y, et al (2002) TGFbeta-Smad signalling in postoperative human lens epithelial cells. Br J Ophthalmol 86: 1428–1433. 12446380 10.1136/bjo.86.12.1428 PMC1771405

[14] Wormstone IM, Tamiya S, Eldred JA, Lazaridis K, Chantry A, et al (2004) Characterisation of TGF-beta2 signalling and function in a human lens cell line. Exp Eye Res 78: 705–714. 15106950 10.1016/j.exer.2003.08.006

[15] Wormstone IM, Anderson IK, Eldred JA, Dawes LJ, Duncan G (2006) Short-term exposure to transforming growth factor beta induces long-term fibrotic responses. Exp Eye Res 83: 1238–1245. 16934251 10.1016/j.exer.2006.06.013

[16] Lovicu FJ, Schulz MW, Hales AM, Vincent LN, Overbeek PA, et al (2002) TGFbeta induces morphological and molecular changes similar to human anterior subcapsular cataract. Br J Ophthalmol 86: 220–226. 11815351 10.1136/bjo.86.2.220 PMC1771017

[17] de Iongh RU, Wederell E, Lovicu FJ, McAvoy JW (2005) Transforming growth factor-beta-induced epithelial-mesenchymal transition in the lens: a model for cataract formation. Cells Tissues Organs 179: 43–55. 15942192 10.1159/000084508

[18] Banh A, Deschamps PA, Gauldie J, Overbeek PA, Sivak JG, et al (2006) Lens-specific expression of TGF-beta induces anterior subcapsular cataract formation in the absence of Smad3. Invest Ophthalmol Vis Sci 47: 3450–3460. 16877415 10.1167/iovs.05-1208 PMC2811063

[19] McDonnell PJ, Zarbin MA, Green WR (1983) Posterior capsule opacification in pseudophakic eyes. Ophthalmology 90: 1548–1553. 6677855 10.1016/s0161-6420(83)34350-5

[20] Gerhart J, Pfautz J, Neely C, Elder J, DuPrey K, et al (2009) Noggin producing, MyoD-positive cells are crucial for eye development. Dev Biol 336: 30–41. 19778533 10.1016/j.ydbio.2009.09.022 PMC2783511

[21] Gerhart J, Elder J, Neely C, Schure J, Kvist T, et al (2006) MyoD-positive epiblast cells regulate skeletal muscle differentiation in the embryo. J Cell Biol 175: 283–292. 17060497 10.1083/jcb.200605037 PMC2064569

[22] Walker JL, Zhai N, Zhang L, Bleaken BM, Wolff I, et al (2010) Unique precursors for the mesenchymal cells involved in injury response and fibrosis. Proc Natl Acad Sci U S A 107: 13730–13735. 20634425 10.1073/pnas.0910382107 PMC2922264

[23] Gerhart J, Baytion M, DeLuca S, Getts R, Lopez C, et al (2000) DNA dendrimers localize MyoD mRNA in presomitic tissues of the chick embryo. J Cell Biol 149: 825–834. 10811824 10.1083/jcb.149.4.825 PMC2174576

[24] Gerhart J, Neely C, Stewart B, Perlman J, Beckmann D, et al (2004) Epiblast cells that express MyoD recruit pluripotent cells to the skeletal muscle lineage. J Cell Biol 164: 739–746. 14981095 10.1083/jcb.200309152 PMC1615912

[25] Gerhart J, Bast B, Neely C, Iem S, Amegbe P, et al (2001) MyoD-positive myoblasts are present in mature fetal organs lacking skeletal muscle. J Cell Biol 155: 381–392. 11684706 10.1083/jcb.200105139 PMC2150848

[26] Gerhart J, Scheinfeld VL, Milito T, Pfautz J, Neely C, et al (2011) Myo/Nog cell regulation of bone morphogenetic protein signaling in the blastocyst is essential for normal morphogenesis and striated muscle lineage specification. Dev Biol 359: 12–25. 21884693 10.1016/j.ydbio.2011.08.007 PMC3192235

[27] Gerhart J, Hayes C, Scheinfeld V, Chernick M, Gilmour S, et al (2012) Myo/Nog cells in normal, wounded and tumor bearing skin. Exp Dermatol 21: 466–468. 22621191 10.1111/j.1600-0625.2012.01503.x PMC3367384

[28] Gerhart J, Neely C, Elder J, Pfautz J, Perlman J, et al (2007) Cells that express MyoD mRNA in the epiblast are stably committed to the skeletal muscle lineage. J Cell Biol 178: 649–660. 17698608 10.1083/jcb.200703060 PMC2064471

[29] Strony R, Gerhart J, Tornambe D, Perlman J, Neely C, et al (2005) NeuroM and MyoD are expressed in separate subpopulations of cells in the pregastrulating epiblast. Gene Expr Patterns 5: 387–395. 15661645 10.1016/j.modgep.2004.09.006

[30] George-Weinstein M, Gerhart J, Reed R, Flynn J, Callihan B, et al (1996) Skeletal myogenesis: the preferred pathway of chick embryo epiblast cells in vitro. Dev Biol 173: 279–291. 8575629 10.1006/dbio.1996.0023

[31] Wormstone IM, Liu CS, Rakic JM, Marcantonio JM, Vrensen GF, et al (1997) Human lens epithelial cell proliferation in a protein-free medium. Invest Ophthalmol Vis Sci 38: 396–404. 9040473

[32] Ishizaki Y, Voyvodic JT, Burne JF, Raff MC (1993) Control of lens epithelial cell survival. J Cell Biol 121: 899–908. 8491781 10.1083/jcb.121.4.899 PMC2119790

[33] Gerhart J, Baytion M, Perlman J, Neely C, Hearon B, et al (2004) Visualizing the Needle in the Haystack: In Situ Hybridization With Fluorescent Dendrimers. Biol Proced Online 6: 149–156. 15272365 10.1251/bpo84 PMC481046

[34] Pinney DF, Pearson-White SH, Konieczny SF, Latham KE, Emerson CP Jr (1988) Myogenic lineage determination and differentiation: evidence for a regulatory gene pathway. Cell 53: 781–793. 3286015 10.1016/0092-8674(88)90095-5

[35] Tonegawa A, Takahashi Y (1998) Somitogenesis controlled by Noggin. Dev Biol 202: 172–182. 9769170 10.1006/dbio.1998.8895

[36] Valenzuela DM, Economides AN, Rojas E, Lamb TM, Nunez L, et al (1995) Identification of mammalian noggin and its expression in the adult nervous system. J Neurosci 15: 6077–6084. 7666191 10.1523/JNEUROSCI.15-09-06077.1995 PMC6577675

[37] Isaacs WB, Cook RK, Van Atta JC, Redmond CM, Fulton AB (1989) Assembly of vimentin in cultured cells varies with cell type. J Biol Chem 264: 17953–17960. 2808358

[38] Webster C, Silberstein L, Hays AP, Blau HM (1988) Fast muscle fibers are preferentially affected in Duchenne muscular dystrophy. Cell 52: 503–513. 3342447 10.1016/0092-8674(88)90463-1

[39] Bader D, Masaki T, Fischman DA (1982) Immunochemical analysis of myosin heavy chain during avian myogenesis in vivo and in vitro. J Cell Biol 95: 763–770. 6185504 10.1083/jcb.95.3.763 PMC2112936

[40] Kintner CR, Brockes JP (1984) Monoclonal antibodies identify blastemal cells derived from dedifferentiating limb regeneration. Nature 308: 67–69. 6366572 10.1038/308067a0

[41] Jin J, Lin J–C, Lin JJ-C (1989) Troponin T isoform switching during heart development. Ann NY Acad Sci 588: 393–396.

[42] Alizadeh A, Clark J, Seeberger T, Hess J, Blankenship T, et al (2003) Targeted deletion of the lens fiber cell-specific intermediate filament protein filensin. Invest Ophthalmol Vis Sci 44: 5252–5258. 14638724 10.1167/iovs.03-0224

[43] Alizadeh A, Clark JI, Seeberger T, Hess J, Blankenship T, et al (2002) Targeted genomic deletion of the lens-specific intermediate filament protein CP49. Invest Ophthalmol Vis Sci 43: 3722–3727. 12454043

[44] Yoon KH, Blankenship T, Shibata B, Fitzgerald PG (2008) Resisting the effects of aging: a function for the fiber cell beaded filament. Invest Ophthalmol Vis Sci 49: 1030–1036. 18326727 10.1167/iovs.07-1149 PMC6746185

[45] Dodd J, Morton SB, Karagogeos D, Yamamoto M, Jessell TM (1988) Spatial regulation of axonal glycoprotein expression on subsets of embryonic spinal neurons. Neuron 1: 105–116. 3272160 10.1016/0896-6273(88)90194-8

[46] Jagla K, Dolle P, Mattei MG, Jagla T, Schuhbaur B, et al (1995) Mouse Lbx1 and human LBX1 define a novel mammalian homeobox gene family related to the Drosophila lady bird genes. Mech Dev 53: 345–356. 8645601 10.1016/0925-4773(95)00450-5

[47] Hales AM, Chamberlain CG, McAvoy JW (1995) Cataract induction in lenses cultured with transforming growth factor-beta. Invest Ophthalmol Vis Sci 36: 1709–1713. 7601651

[48] de Iongh RU, Lovicu FJ, Overbeek PA, Schneider MD, Joya J, et al (2001) Requirement for TGFbeta receptor signaling during terminal lens fiber differentiation. Development 128: 3995–4010. 11641223 10.1242/dev.128.20.3995

[49] Dawes LJ, Eldred JA, Anderson IK, Sleeman M, Reddan JR, et al (2008) TGF beta-induced contraction is not promoted by fibronectin-fibronectin receptor interaction, or alpha SMA expression. Invest Ophthalmol Vis Sci 49: 650–661. 18235011 10.1167/iovs.07-0586

[50] Sharma N, Pushker N, Dada T, Vajpayee RB, Dada VK (1999) Complications of pediatric cataract surgery and intraocular lens implantation. J Cataract Refract Surg 25: 1585–1588. 10609200 10.1016/s0886-3350(99)00296-5

[51] Baratz KH, Cook BE, Hodge DO (2001) Probability of Nd:YAG laser capsulotomy after cataract surgery in Olmsted County, Minnesota. Am J Ophthalmol 131: 161–166. 11228290 10.1016/s0002-9394(00)00795-9

[52] Boureau C, Lafuma A, Jeanbat V, Smith AF, Berdeaux G (2009) Cost of cataract surgery after implantation of three intraocular lenses. Clin Ophthalmol 3: 277–285. 19668579 10.2147/opth.s4890 PMC2708987

[53] Apple DJ, Solomon KD, Tetz MR, Assia EI, Holland EY, et al (1992) Posterior capsule opacification. Surv Ophthalmol 37: 73–116. 1455302 10.1016/0039-6257(92)90073-3

[54] Findl O, Buehl W, Bauer P, Sycha T (2007) Interventions for preventing posterior capsule opacification. Cochrane Database Syst Rev: CD003738. 10.1002/14651858.CD003738.pub3 PMC10658648 20166069

[55] Bertelmann E, Kojetinsky C (2001) Posterior capsule opacification and anterior capsule opacification. Curr Opin Ophthalmol 12: 35–40. 11150079 10.1097/00055735-200102000-00007

[56] Hay ED (1995) An overview of epithelio-mesenchymal transformation. Acta Anat (Basel) 154: 8–20. 8714286 10.1159/000147748

[57] Lim J, Thiery JP (2012) Epithelial-mesenchymal transitions: insights from development. Development 139: 3471–3486. 22949611 10.1242/dev.071209

[58] Nieto MA (2011) The ins and outs of the epithelial to mesenchymal transition in health and disease. Annu Rev Cell Dev Biol 27: 347–376. 21740232 10.1146/annurev-cellbio-092910-154036

[59] Rock JR, Barkauskas CE, Cronce MJ, Xue Y, Harris JR, et al (2011) Multiple stromal populations contribute to pulmonary fibrosis without evidence for epithelial to mesenchymal transition. Proc Natl Acad Sci U S A 108: E1475–1483. 22123957 10.1073/pnas.1117988108 PMC3248478

[60] Kriz W, Kaissling B, Le Hir M (2011) Epithelial-mesenchymal transition (EMT) in kidney fibrosis: fact or fantasy? J Clin Invest 121: 468–474. 21370523 10.1172/JCI44595 PMC3026733

[61] Barth PJ, Westhoff CC (2007) CD34+ fibrocytes: morphology, histogenesis and function. Curr Stem Cell Res Ther 2: 221–227. 18220905 10.2174/157488807781696249

[62] Gabbiani G (2003) The myofibroblast in wound healing and fibrocontractive diseases. J Pathol 200: 500–503. 12845617 10.1002/path.1427

[63] De Wever O, Demetter P, Mareel M, Bracke M (2008) Stromal myofibroblasts are drivers of invasive cancer growth. Int J Cancer 123: 2229–2238. 18777559 10.1002/ijc.23925

[64] Sappino AP, Schurch W, Gabbiani G (1990) Differentiation repertoire of fibroblastic cells: expression of cytoskeletal proteins as marker of phenotypic modulations. Lab Invest 63: 144–161. 2116562

[65] Schaart G, Pieper FR, Kuijpers HJ, Bloemendal H, Ramaekers FC (1991) Baby hamster kidney (BHK-21/C13) cells can express striated muscle type proteins. Differentiation 46: 105–115. 2065865 10.1111/j.1432-0436.1991.tb00871.x

[66] Ogata I, Saez CG, Greenwel P, Ponce Mde L, Geerts A, et al (1993) Rat liver fat-storing cell lines express sarcomeric myosin heavy chain mRNA and protein. Cell Motil Cytoskeleton 26: 125–132. 8287498 10.1002/cm.970260204

[67] Mayer DC, Leinwand LA (1997) Sarcomeric gene expression and contractility in myofibroblasts. J Cell Biol 139: 1477–1484. 9396753 10.1083/jcb.139.6.1477 PMC2132619

[68] Woodcock-Mitchell J, Mitchell JJ, Low RB, Kieny M, Sengel P, et al (1988) Alpha-smooth muscle actin is transiently expressed in embryonic rat cardiac and skeletal muscles. Differentiation 39: 161–166. 2468547 10.1111/j.1432-0436.1988.tb00091.x

[69] Michael R, Vrensen GF, van Marle J, Lofgren S, Soderberg PG (2000) Repair in the rat lens after threshold ultraviolet radiation injury. Invest Ophthalmol Vis Sci 41: 204–212. 10634622

[70] Maddala R, Deng PF, Costello JM, Wawrousek EF, Zigler JS, et al (2004) Impaired cytoskeletal organization and membrane integrity in lens fibers of a Rho GTPase functional knockout transgenic mouse. Lab Invest 84: 679–692. 15094715 10.1038/labinvest.3700105

[71] Andjelic S, Zupancic G, Perovsek D, Hawlina M (2011) Human anterior lens capsule epithelial cells contraction. Acta Ophthalmol 89: e645–653. 21801334 10.1111/j.1755-3768.2011.02199.x

